# Identification of enterotype and its predictive value for patients with colorectal cancer

**DOI:** 10.1186/s13099-024-00606-y

**Published:** 2024-02-27

**Authors:** Li Qingbo, Zhuang Jing, Qu Zhanbo, Chu Jian, Song Yifei, Wu Yinhang, Han Shuwen

**Affiliations:** 1 Huzhou Central Hospital, Affiliated Central Hospital Huzhou University, Huzhou, Zhejiang Province, People’s Republic of China; 2 Fifth School of Clinical Medicine of Zhejiang Chinese Medical University, Huzhou, Zhejiang Province, People’s Republic of China; 3 Key Laboratory of Multiomics Research and Clinical Transformation of Digestive Cancer, No.1558, Sanhuan North Road, Wuxing District, Huzhou, Zhejiang Province 313000 People’s Republic of China

**Keywords:** Enterotype, Adenoma, Colorectal cancer, Gut microbiota, Random forest model

## Abstract

**Background:**

Gut microbiota dysbiosis involved in the pathogenesis of colorectal cancer (CRC). The characteristics of enterotypes in CRC development have not been determined.

**Objective:**

To characterize the gut microbiota of healthy, adenoma, and CRC subjects based on enterotype.

**Methods:**

The 16 S rRNA sequencing data from 315 newly sequenced individuals and three previously published datasets were collected, providing total data for 367 healthy, 320 adenomas, and 415 CRC subjects. Enterotypes were analyzed for all samples, and differences in microbiota composition across subjects with different disease states in each enterotype were determined. The predictive values of a random forest classifier based on enterotype in distinguishing healthy, adenoma, and CRC subjects were evaluated and validated.

**Results:**

Subjects were classified into one of three enterotypes, namely, *Bacteroide*- (BA_E), *Blautia*- (BL_E), and *Streptococcus*- (S_E) dominated clusters. The taxonomic profiles of these three enterotypes differed among the healthy, adenoma, and CRC cohorts. BA_E group was enriched with *Bacteroides* and *Blautia*; BL_E group was enriched by *Blautia* and *Coprococcus*; S_E was enriched by *Streptococcus* and *Ruminococcus.* Relative abundances of these genera varying among the three human cohorts. In training and validation sets, the S_E cluster showed better performance in distinguishing among CRC patients, adenoma patients, and healthy controls, as well as between CRC and non-CRC individuals, than the other two clusters.

**Conclusion:**

This study provides the first evidence to indicate that changes in the microbial composition of enterotypes are associated with disease status, thereby highlighting the diagnostic potential of enterotypes in the treatment of adenoma and CRC.

**Supplementary Information:**

The online version contains supplementary material available at 10.1186/s13099-024-00606-y.

## Highlights

Three enterotypes (BA_E, BL_E, and S_E) were identified in healthy, adenoma, and CRC subjects.

BA_E, BL_E, and S_E clusters were dominated by *Bacteroide*, *Blautia*, and *Streptococcus*, respectively.

Differences in gut microbial composition were observed within the control, adenoma, CRC populations for each enterotype.

S_E showed better performance in distinguishing three human cohorts than BA_E and BL_E.

Disease prediction performance of enterotypes is no better than that of a classification model based on all samples.

**Supplementary Information:**

The online version contains supplementary material available at 10.1186/s13099-024-00606-y.

## Introduction

The human gastrointestinal tract is inhabited by a community of approximately 100 trillion bacteria, viruses, and fungi that provide the host with unique metabolic functions and influence human health and diseases [1]. In humans, a healthy gut microbiota plays essential roles in shaping the intestinal epithelium, obtaining energy, maintaining immunity, and defending against pathogens [2–4]. Conversely, dysbiosis of the intestinal microbiome can alter host physiological functions and result in a number of diseases and disorders [5]. Consequently, the intestinal microbiome may influence the development of chronic diseases, ranging from metabolic disorders such as type 2 diabetes to gastrointestinal disorders and colorectal cancer (CRC) [6, 7].

CRC continues to remain a prominent global health burden, and the latest epidemiological surveys reveal that the incidence of early-onset CRC is increasing year by year, accompanied by poor prognoses [8]. Colorectal adenoma is the major precancerous lesion of CRC, accounting for 85–90% of all CRC precancerous diseases [9]. In addition, most CRC cases are sporadic and develop primarily via an adenoma–carcinoma sequence [10]. The gut microbiota has been identified as a key factor contributing to CRC development, and several specific intestinal bacteria, such as *Fusobacterium nucleatum* and the enterotoxigenic *Bacteroides fragilis*, may be involved in the development of adenoma and subsequent colorectal carcinogenesis [11]. Moreover, reductions in the relative abundance of certain microbial taxa such as *Faecalibacterium* and *Bacteroides* have previously been detected in adenomatous polyp and oncogenic mucosal samples, thereby indicating that these bacteria could serve as potential novel biomarkers for the detection of early carcinogenesis [12]. These studies thus highlight the importance of investigating changes in the gut microbiota during progression of the adenoma–carcinoma sequence to characterize CRC pathogenesis and screening for early diagnostic markers.

The classification of enterotype based on gut microbes is helpful to elucidate the symbiotic relationship between host and microbe. Arumugam et al. [13] analyzed fecal metagenomic data obtained for individuals from six different countries and determined three robust clusters (also referred to enterotypes), without national or continental specificity. The three enterotypes are driven by preferred species in each community composition, including *Bacteroides*, *Prevotella*, and *Ruminococcus*. It has been established that specific enterotypes are associated with long-term dietary habits and that those individual with different enterotypes have different ways of metabolizing and storing energy [14]. Furthermore, there is accumulating evidence to indicate that enterotypes play important roles in driving multiple pathophysiological processes [15, 16]. Accordingly, it is argued that if patients could be grouped in terms enterotype, this would make an important contribution to realizing personalized microbiome-based diagnosis and treatment in clinical practice. For example, Yang et al. [17] performed an enterotypes analysis of the fecal microbiota from patients with CRC and revealed that the dysbiosis characteristics of the CRC gut microbiota differed according to enterotype. In addition, a high abundance of the *Prevotella* enterotype may affect the development of CRC by regulating the expression of immune response-related genes in the intestinal mucosa [18]. These and similar findings indicate that enterotypes may constitute an important microbial characteristic of CRC. Given that the normal adenoma–cancer sequence model reflects the evolution of colorectal carcinogenesis, the different compositions of enterotypes in the three populations would appear to be important for understanding the mechanisms of CRC development. However, relatively few studies have focused on and compared the dysbiosis characteristics of the gut microbiota in adenoma and CRC patients with different enterotypes.

In this study, our objective was to focus on the similarities and differences in the gut microbiota among healthy, adenoma, and CRC subjects from the perspective of enterotypes. By integrating 16 S rRNA sequencing data from 315 clinical samples with those of three previously published datasets (a total of 1102 samples), the gut microbiota characteristics of patients in healthy, adenoma, and CRC based on different enterotypes were analyzed. Furthermore, a random forest classification method was used to construct a model that could be used to discriminate among the three human cohorts. The findings of this research provide a novel perspective for further revealing the predictive role of microbial enterotypes in the occurrence and development of CRC.

## Materials and methods

### Human subjects and sample collection

The subjects assessed in this study were patients with either adenoma or CRC and healthy volunteers who visited Huzhou Central Hospital from 2020 to 2021. Patients with adenoma and CRC were confirmed by pathological examination. Participant exclusion criteria were as follows: [1] patients with other malignancies, [2] patients with severe cardiopulmonary disease, [3] patients with a history of oral intestinal flora preparation 1 month prior to admission, and [4] patients with other intestinal diseases, such as ulcerative colitis and Crohn’s disease. Finally, 315 participants were included in this study, among whom, 28 had adenomas, 202 had CRC, and 85 were healthy controls. Fresh fecal sample from each subject was collected and immediately stored at -80 °C until sequenced.

All procedures involving human participants were performed in accordance with the standards of the ethics committee of Huzhou Central Hospital and the Declaration of Helsinki (1964). Meanwhile, informed consent was obtained from all participants enrolled in this study.

### DNA extraction and 16 S rRNA sequencing

Microbial DNA was extracted from frozen fecal samples using a QIAamp DNA Stool Mini kit and the integrity and size of the extracted DNA were examined using 1% agar gel electrophoresis. Subsequently, PCR amplification was performed on the v3-v4 region of the 16 S rRNA gene using previously described PCR primers and reaction conditions [19]. The PCR products this obtained were detected and quantified using a QuantiFluor™ -ST Blue Fluoror quantification System (Promega). Thereafter, sequencing libraries were generated using a TruSeqTM DNA Sample Prep Kit and sequenced using the Illumina MiSeq platform (paired-end approach).

### Dataset sources and data preprocessing

To increase the sample size for analysis, we also collected the gut microbiota data of healthy, adenoma, and CRC subjects from the GMrepo (https://gmrepo.humangut.info) [20] and NCBI (https://www.ncbi.nlm.nih.gov/sra) [21] databases, and three datasets with accession numbers PRJEB6070 [22], PRJNA280026 [23], and PRJNA290926 [24] were selected. The 16 S rRNA sequencing data of three datasets and newly sequenced clinical samples were analyzed using QIIME2 [25], and the reads were subsequently denoised using DADA2 [26] to obtain amplicon sequence variant (ASV) table. For 16 S data, QIIME2 version 2021.2 pipeline was selected. After matching with the reference database Greengenes (v.13.8), the species composition based on genus level was obtained for further analysis. Samples with less than five microbial species were removed. Besides, to avoid noises caused by low abundant taxa, samples with relative abundance < 0.001 were deleted. Detailed quality control steps refer to the article of Dai D et al. [27] .Thus, a total of 1102 samples were obtained and used as a training cohort. The specific numbers of samples for each of the three public datasets were as follows: project PRJEB6070 data included those for 38 adenoma, 41 CRC, and 50 healthy individuals from France; project PRJNA280026 data included those for 56 adenoma, 52 CRC, and 60 healthy individuals from China; and project PRJNA290926 data included those for 198 adenoma, 120 CRC, and 172 healthy individuals from the USA.

Furthermore, to validate our findings, as external validation cohorts, we used three whole-genome sequencing (WGS) datasets (accession numbers PRJEB10878, PRJEB27928, and PRJDB4176) obtained from the GMrepo database. Metagenomic data of patients with CRC in the three datasets were downloaded from GMrepo, and samples with detailed country and age information were screened, whereas samples with apparently incorrect information such as body mass index and age were excluded. All WGS sequencing data were annotated using MetaPhlAn2 [28] to obtain the relative abundance of species composition at the genus level for further analysis. There are different species in the same genus. If the proportion of this species is less than 2%, then this species will be excluded. Finally, a total of 286 samples were included. The PRJDB4176 dataset contained 40 CRC and 40 healthy subjects from Japan; the PRJEB10878 dataset contained 72 CRC and 54 healthy subjects from China; PRJEB27928 dataset contained 21 CRC and 59 healthy subjects from Germany.

### Identification of enterotypes

The clusterSim package in cluster was used to analyze enterotypes of 1102 samples. Briefly, using the genus-level data, we initially calculated the Jensen Shannon divergence (JSD) between sample, after which the Partitioning Around Medoids algorithm was applied to cluster the samples, and the optimal number of clusters was determined based on the Calinski–Harabasz (CH) index, silhouette width、Davies-Bouldin (DBI) index and Dunnindex. Except for DBI, the maximum index is selected as the optimal cluster number. Finally, we obtained three prominent clusters. In addition, the DMM method was selected to verify the clustering effect. Species selection was performed using the resting function of DirichletMultinomial packets. The minimum abundance threshold was set to 0.01% and the minimum persistence was set to 1%. Furthermore, principal coordinate analysis (PCoA) of the JSD matrix was performed using the ade4 package, and the dominant bacteria (top 8) of each enterotype were visualized using the ggolot2 package.

### Analysis of sample composition in different enterotypes

The three identified enterotypes were grouped according to three different disease states (adenoma, CRC, and healthy). Genera were comprehensively characterized based on species prevalence and mean abundance, with the top 20 genera in each group being displayed using ggolot2. Moreover, MicrobiotaProcess package was used to plot the principal component analysis (PCA) of the logarithms of species relative abundance.

### Differential analysis

Using the enterotype data, the microbiomeMarker package was applied to evaluate differences in the gut microbiota in subjects with one of the three disease states, and genera identified by an effect size linear discriminant analysis (LDA) score > 2 were considered to be differentially enriched taxa. In addition, to validate the enterotype clustering results, we also performed bacterial differential analysis for the three human cohorts using on samples without a differentiation of enterotypes. Besides, integration of biomarkers from the three enterotypes and all samples was performed using the VennDiagram package.

### Correlation analysis

For individuals characterized by different disease status, we calculated SECOM correlation coefficient coefficient (r) values between the relative abundance of the differential species for each of the three enterotypes by using ANCOMBC package. Species with an |r| value > 0.3 and p value < 0.05 were retained, and the remaining species were assigned a zero value. Corresponding correlation heatmaps were plotted using the corrplot package.

### Construction of random forest classifier models

For each of the three enterotypes, as well as for all samples without differentiation of enterotypes, we constructed three-class (healthy/adenoma/CRC) and two-class (non-CRC/CRC) classification models using the random forest method. Briefly, models were developed using the randomForest and caret packages, and the accuracy of the trained classifier was evaluated via three rounds of nested 5-fold cross-validation. Thereafter, the multiClassSummary function was applied to calculate the evaluation index of classification (F1 score, sensitivity, and specificity) and the pROC package was used to assess area under the curve (AUC) values. In addition, we used the ggplot2 package to extract and visualize the top 20 features of the random forest model in terms of average importance, and the three validation datasets were utilized to verify the predictive performance of the constructed models.

## Results

### Overall characteristic of microbial communities in the three enterotypes

**Fig. 1:**
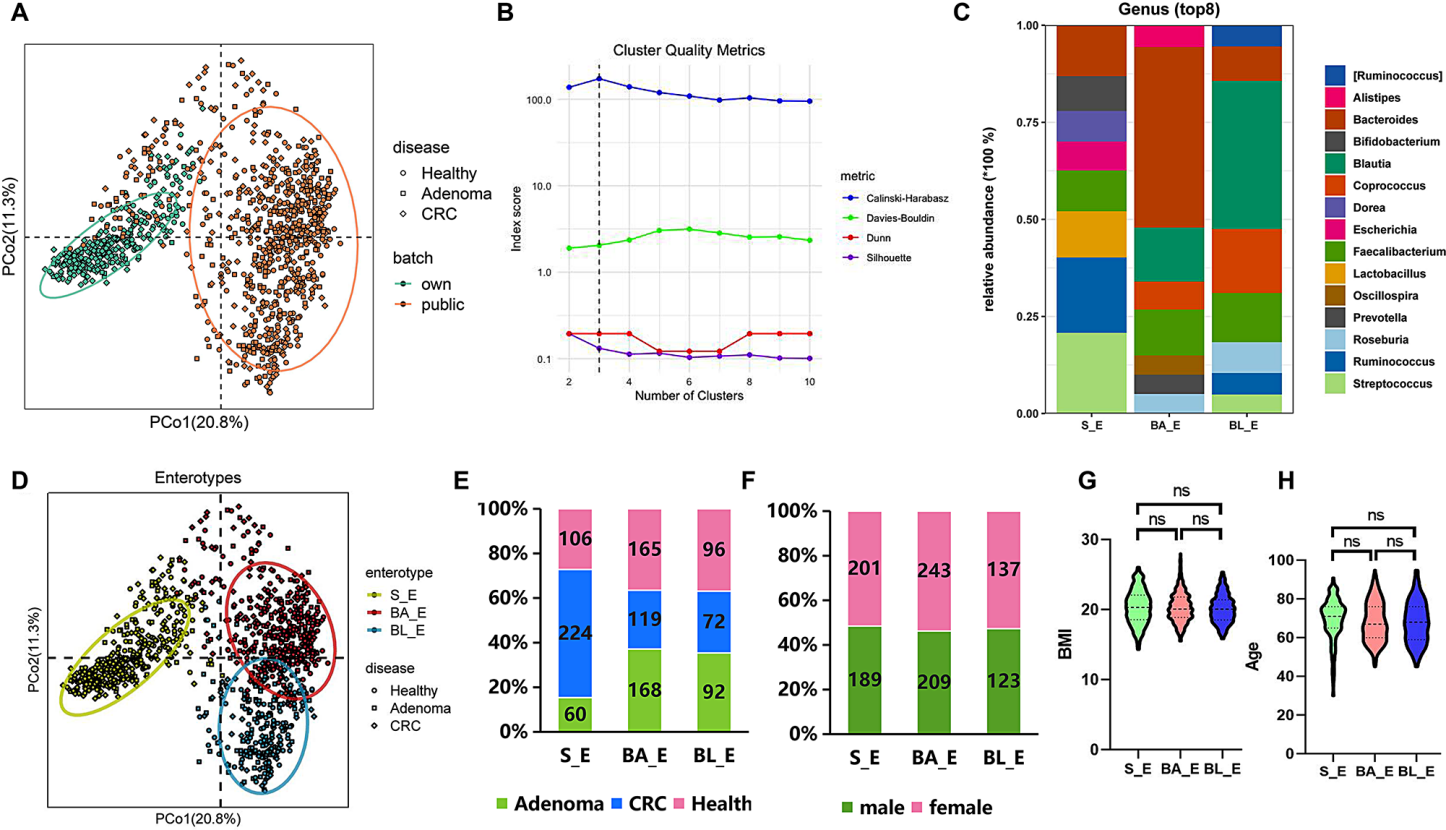
Gut enterotype analysis of 1102 samples. **A**: The source of the data (own vs. public) coloring by a simple PCoA plot. **B**: Calinski–Harabasz (CH) index analysis based on the Jensen Shannon divergence (JSD) distance. K = 3 is the optimal number of clusters. **C**: The top eight genera in each enterotype. **D**: Principal coordinates analysis (PCoA) plot of the three enterotypes. All samples (adenoma, *n* = 320; CRC, *n* = 415; healthy, *n* = 367) are clustered into “*Streptococcus*” (S_E, green), “*Bacteroide*” (BA_E, red), and “*Blautia*” (BL_E, blue) enterotypes. E: A bar chart showing the distribution of different disease states in three enterotypes. Red, green, and blue represent healthy control, adenoma, and blue CRC samples, respectively. F-H: The distribution of clinical factors such as sex, BMI and age among the three enterotype. ns meant no statistical difference between the two groups (*P* > 0.05)

First, the source of the data (own vs. public) were analyzed by PCoA plot (Fig. 1A). Then, 1102 samples were clustered according to the relative abundance of bacteria at the genus level using the JSD distance metric. Considering the silhouette width, CH index, DBI index, and Dunn index, three clusters are determined when K = 3 (Fig. 1B). Based on the dominant bacteria in each group, the three enterotypes were respectively designated *Streptococcus* (S_E, *n* = 390), *Bacteroide* (BA_E, *n* = 452), and *Blautia* (BL_E, *n* = 260). The eight most abundant genera in each enterotype are shown in Fig. 1C. In brief, *Streptococcus* (23.02%) and *Ruminococcus* (14.52%) were relatively abundant in S_E type. *Bacteroides* (46.51%) and *Blautia* (13.91%) were relatively abundant in BA_E type; *Blautia* (35.87%) and *Coprococcus* (15.50%)were relatively abundant in BL_E group. A PCoA plot confirmed the differences among the three enterotypes, while the BA_E and BL_E enterotypes showed a certain degree of overlap (Fig. 1D). In addition, we observed the number of people with different health states in each enterotype (Fig. 1E). The S_E group included 106 healthy, 60 adenoma, and 224 CRC subjects; the BA_E group included 165 healthy, 168 adenoma, and 119 CRC subjects; the BL_E group included 96 healthy, 92 adenoma, and 72 CRC subjects. Thus, compared with the BA_E and BL_E enterotypes, the S_E group comprised the highest proportion of patients with CRC (57% vs. 26% and 28%). The proportions of adenoma and healthy subjects with the BA_E and BL_E enterotypes were essentially the same (35–37%) and somewhat higher than those of the patients with CRC (26–28%). Furthermore, the distribution of clinical factors such as age, sex, and BMI among three enterotypes was also analyzed. The results showed that there were no statistically significant differences in these clinical factors between the three enterotypes (*P* > 0.05) (Fig. 1F-H). Figure S1 shows the top eight bacterial genera in the three enterotypes for each of the subject populations, which indicates that even for subjects in the same population, there were notable differences among the three enterotypes with respect to gut microbiota composition.

### Gut microbiota composition in BA_E for three human cohorts at the genus level

**Fig. 2:**
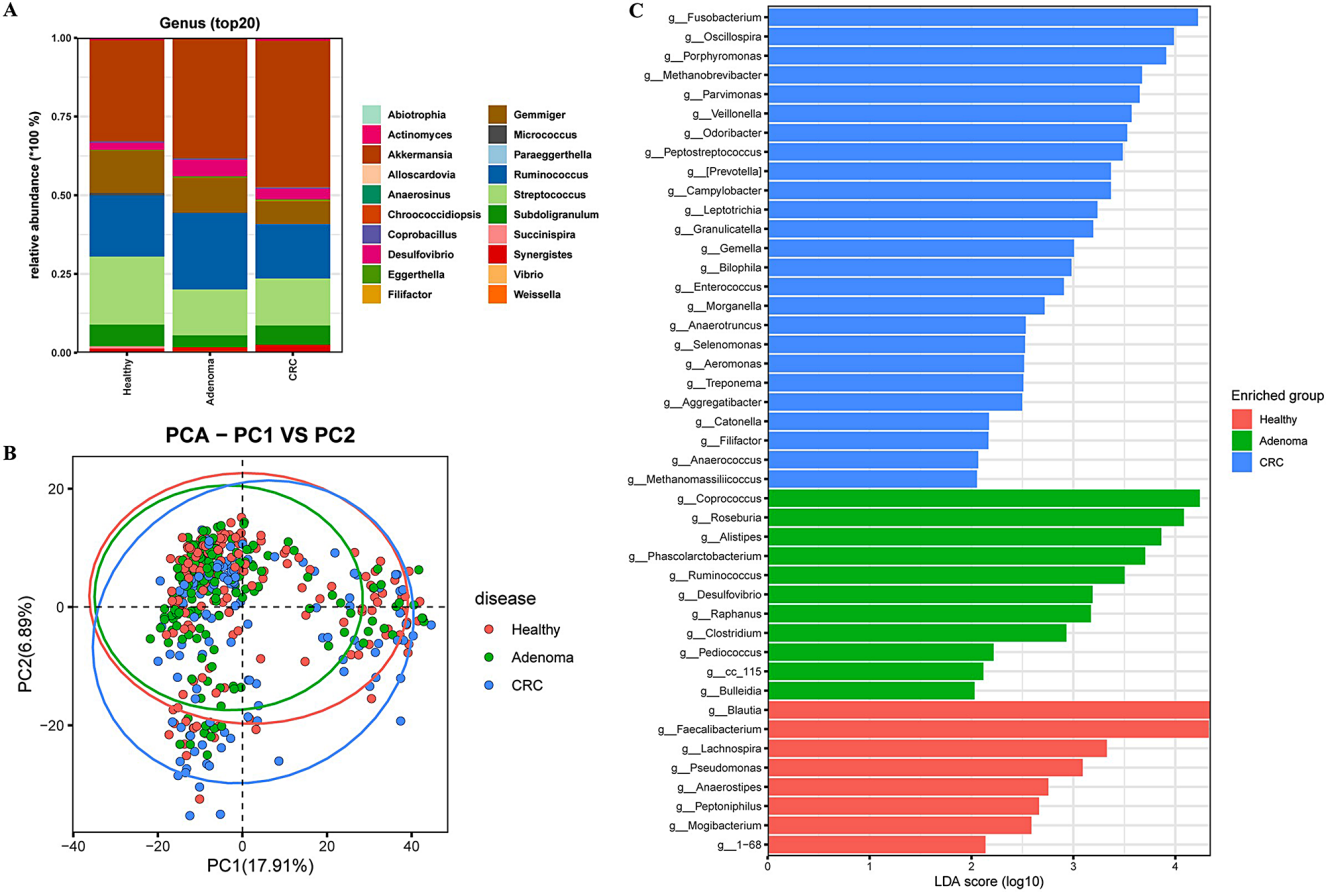
Distinct bacterial composition of samples from healthy subjects, adenoma patients, and colorectal cancer (CRC) patients in the BA_E enterotype. **A**: The community abundance of gut microbiota at the genus level. **B**: Principal component analysis (PCA) plot visualizing the three human cohorts. Red, green, and blue dots represent healthy control, adenoma, and CRC samples, respectively. **C**: Linear discriminant analysis (LDA) identified the differentially abundant genera among healthy, adenoma, and CRC samples

In the BA_E type, the bacterial composition at the genus level of the three human cohorts is shown in Fig. 2A (top 20 genera). Among the three groups, *Akkermansia*, *Ruminococcus*, *Streptococcus*, *Gemmiger*, and *Subdoligranulum* were identified as the five predominant genera. PCA plot indicated that the colony composition of the three populations was not significantly distinguished (Fig. 2B). In further analysis, LDA was used to screen for differences among the three subject populations with respect microbial community species, revealed 44 genera that differed among healthy, adenoma, and CRC subjects (Fig. 2C). Among these, eight genera, including *Blautia*, *Faecalibacterium*, and *Lachnospira*, were significantly enriched in the healthy group; 11 genera, including *Coprococcus*, *Roseburia*, and *Alistipes*, were significantly enriched in the adenoma group; 25 genera, including *Fusobacterium*, *Oscillospira*, and *Porphyromonas*, were significantly enriched in the CRC group.

For each human cohort, we also examined correlations among the differential bacterial genera. In the healthy group (Figure S2A), *Blautia* showed negative correlation with *Lachnospira* (*r* = -0.044), while it was positively correlated with Selenomonas (*r* = 0.008). In the adenoma group (Figure S2B), *Peptostreptococcus* showed positive association with *Parvimonas* (*r* = 0.017) and negative association with *Anaerostipes* (*r* = -0.379). In the CRC group (Figure S2C), *Leptotrichia* was negatively correlated with *Blautia* (*r* = -0.225) and positive association with *Raphanus* (*r* = 0.211).

### Gut microbiota composition in BL_E for three human cohorts at the genus level

**Fig. 3:**
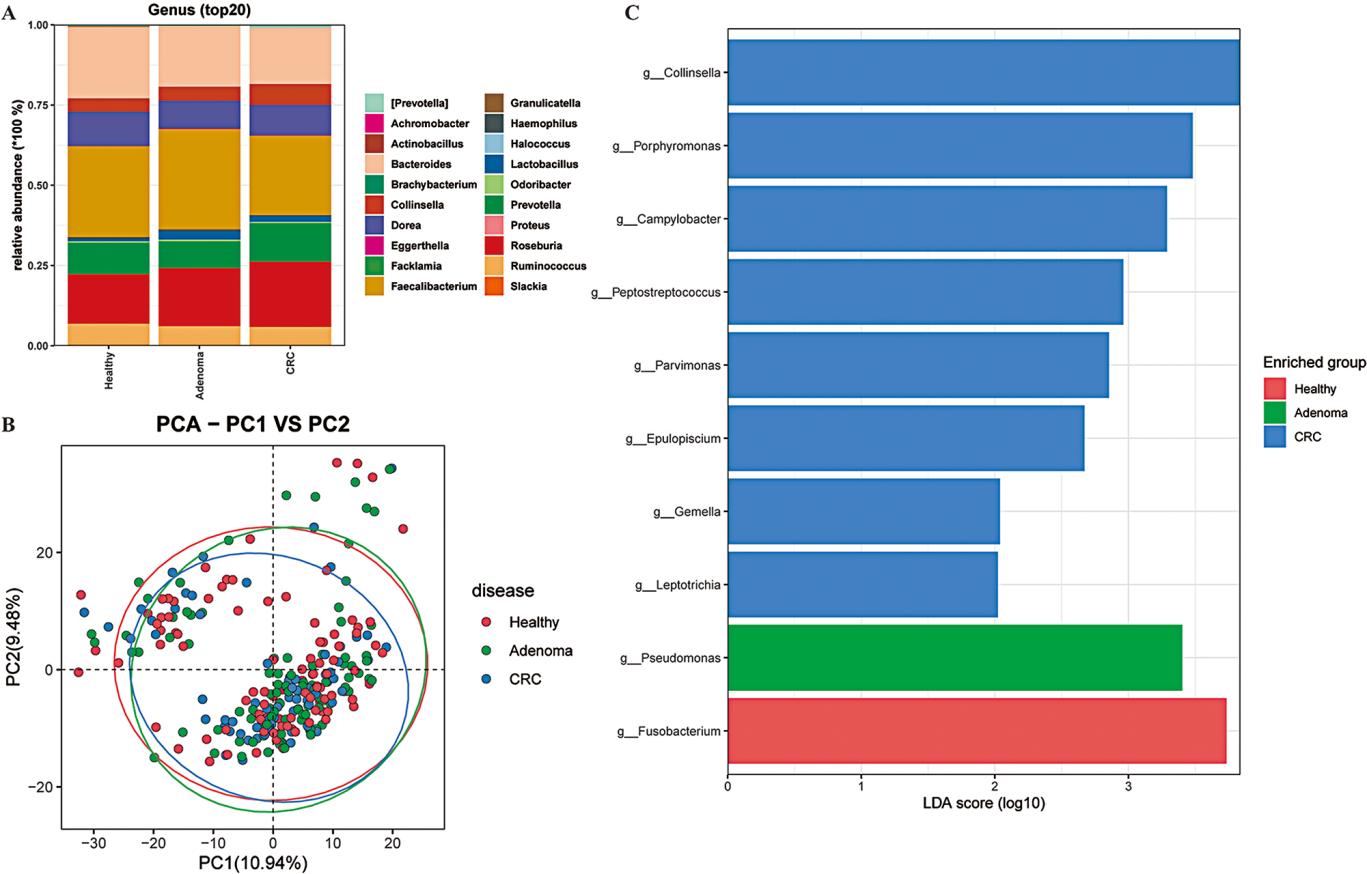
Distinct bacterial composition of samples from healthy subjects, adenoma patients, and CRC patients in the BL_E enterotype. **A**: The community abundance of gut microbiota at the genus level. **B**: PCA plot visualizing the three human cohorts. **C**: LDA identified the differentially abundant genera among the healthy, adenoma, and CRC samples

In the BL_E type, the relative abundances of the top 20 genera for three human cohorts are shown in Fig. 3A. In all three groups, *Faecalibacterium*, *Roseburia*, *Bacteroides*, *Prevotella*, and *Dorea* were the five predominant bacterial genera. PCoA plot showed that the three groups cannot be significantly separated (Fig. 3B). In addition, LDA method was employed to screen the specific genera for each group (Fig. 3C). Briefly, only one genus was significantly enriched in the healthy (*Fusobacterium*) and adenoma (*Pseudomonas*) groups. Eight genera were mainly identified in the CRC group, such as *Collinsella*, *Porphyromonas*, and *Campylobacter*.

Next, we explored the correlation between differential gut microbiota of healthy, adenoma, and CRC samples, respectively. In the healthy group, *Collinsella* exhibited significantly positive correlation with *Peptostreptococcus* (*r* = 0.391), while it was negatively correlated with *Pseudomonas* (*r* = -0.822) (Figure S3A). In the adenoma group, *Collinsella* showed a significantly positive association with *Parvimonas* (*r* = 1), while it was negatively correlated with *Pseudomonas* (*r* = -0.883) and *Fusobacterium* (*r* = -0.857) (Figure S3B). In the CRC group, *Gemella* had strong positive correlation with *Campylobacter* (*r* = 1), and *Peptostreptococcus* had negative correlation with *Pseudomonas* (*r* = -0.578) (Figure S3C).

### Gut microbiota composition in S_E for three human cohorts at the genus level

**Fig. 4:**
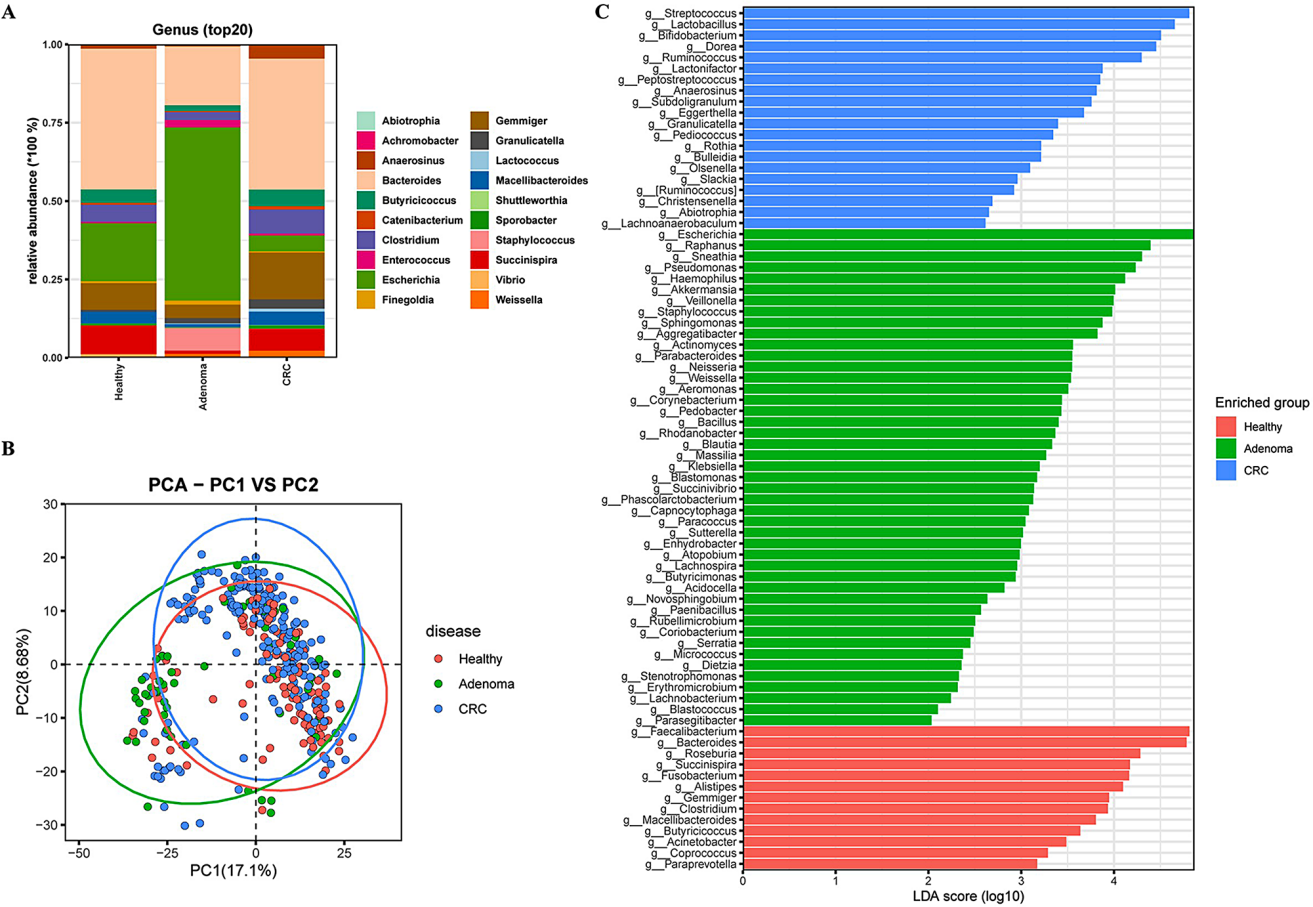
Distinct bacterial composition of samples from healthy subjects, adenoma patients, and CRC patients in the S_E enterotype. **A**: The community abundance of gut microbiota at the genus level. **B**: PCA plot visualizing the three human cohorts. **C**: LDA identified the differentially abundant genera among healthy, adenoma, and CRC samples

Further, the composition of gut microbiota at the genus level in the S_E type was analyzed to describe specific changes in gut microbiota in different disease groups (Fig. 4A). For each of these populations, *Bacteroides*, *Sporobacter*, *Gemmiger*, and *Succinispira* were identified as the predominant bacterial genera. Compared with the healthy and adenoma groups, we observed higher relative abundances of *Gemmiger*, *Clostridium*, and *Anaerosinus* in CRC group patients In contrast, CRC group patients were characterized by the lowest relative abundance of *Escherichia* species, the abundances of which were notably higher in the adenoma group. According to the PCA plot, there was no significant structural differences in gut microbiota among the three groups, while a trend of segregation was observed between adenoma and CRC (Fig. 4B). LDA revealed a total of 78 predominant genera among the three groups, of which 13, 45, and 20 were detected in the healthy, adenoma, and CRC groups respectively (Fig. 4C). Among these, the genera *Faecalibacterium*, *Bacteroides*, and *Roseburia* were identified as dominant bacteria in healthy group; *Escherichia*, *Raphanus*, and *Sneathia* predominated the bacterial community in the adenoma group; *Streptococcus*, *Lactobacillus*, and *Bifidobacterium* were among the predominant genera in the CRC group.

Next, we explored the correlation between 46 differential gut microbiota of healthy, adenoma, and CRC samples, respectively. In the healthy group, *Faecalibacterium* was negatively correlated with *Peptostreptococcus* (*r* = -0.786), while it was positively correlated with *Roseburia* (*r* = 0.366) (Figure S4A). In the adenoma group, *Streptococcus* showed positively correlation with *Pseudomonas* (*r* = 0.4), while it was negatively correlated with *Bifidobacterium* (*r* = -0.8) (Figure S4B). In the CRC group, *Ruminococcus* was found to be significantly negatively correlated with *Bacteroides* ( *r* = -0.335) and *Actinomyces* (*r* = -0.489). *Alistipes* was also found to have a strong positive relationship with *Haemophilus* and *Abiotrophia* (all *r* = 1), while it was negatively with *Actinomyces* (*r* = -0.587) (Figure S4C).

**Fig. 5:**
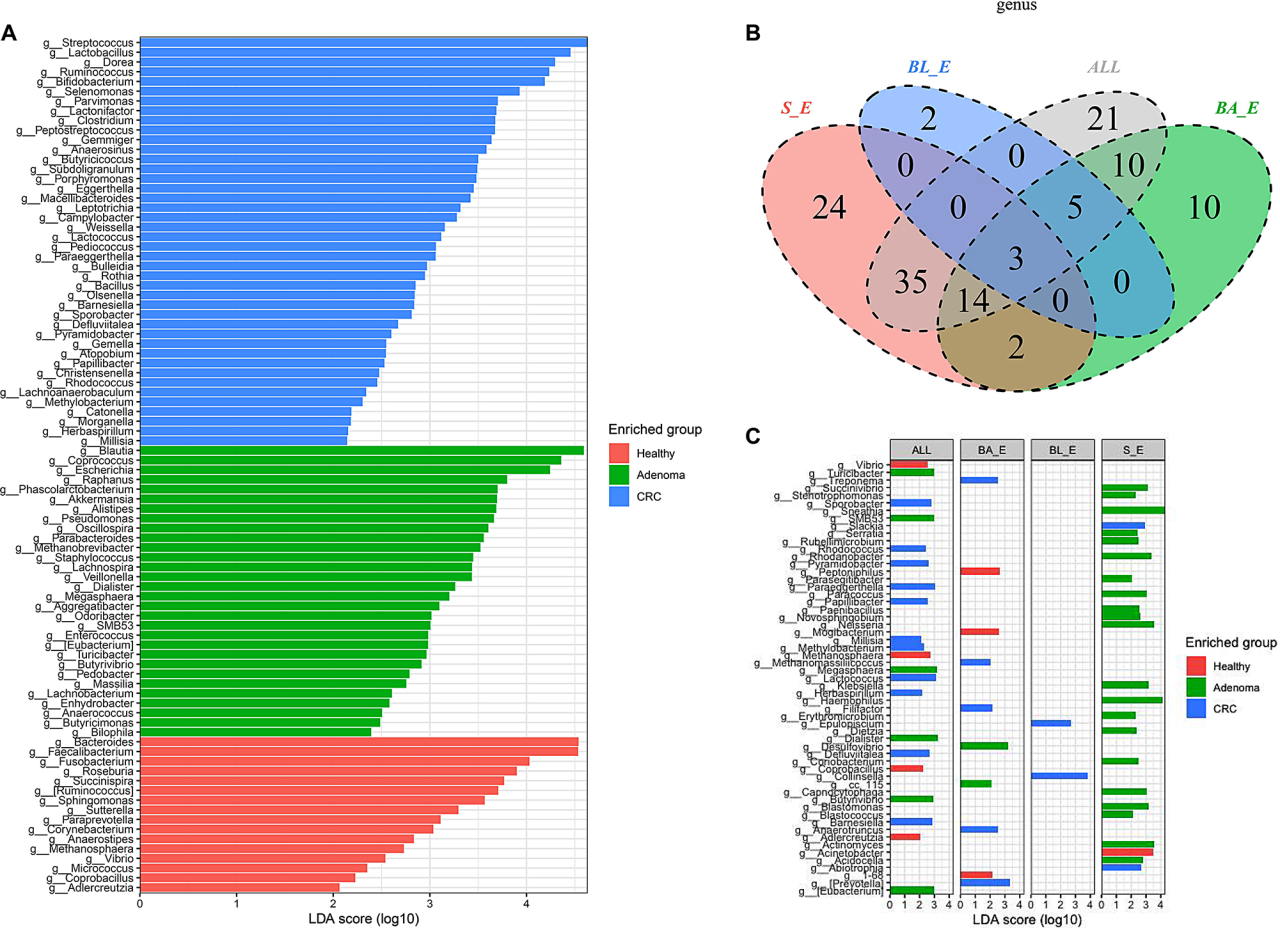
The difference in gut microbiota profiles among healthy subjects, adenoma patients, and CRC patients based on all samples. **A**: LDA identified the differentially abundant genera among healthy, adenoma, and CRC. Red, green, and blue represent healthy control, adenoma, and blue CRC samples, respectively. **B**: A Venn diagram showing the overlap of microbiota within three enterotypes and all samples. Blue, green, and red indicate the BL_E, BA_E, and S_E enterotypes, respectively, and gray indicates all samples. **C**: Different species of each enterotypes screened by LDA analysis

To verify the classification criteria of the three enterotypes, we also analyzed all samples in different disease states without performing enterotype. The results revealed that the three subject groups differed with respect to 89 genera of gut microbiota (Fig. 5A). Briefly, 17 bacterial genera, including *Bacteroides*, *Faecalibacterium*, and *Fusobacterium*, were significantly enriched in the healthy group, whereas 30 bacterial biomarkers, including *Blautia*, *Coprococcus*, and *Escherichia*, were significantly enriched in the adenoma group, and 42 biomarkers, including *Streptococcus*, *Lactobacillus*, and *Dorea*, showed highest abundance in the CRC group. In addition, different species of each enterotypes were screened through Venn analysis and LDA analysis. The results showed that there were 21 specific genera (*Vibrio*, *Turicibacter*, *Sporobacter* and etc.) in all samples, 10 specific genera (*Treponema*, *Peptoniphilus*, *Mogibactenum* and etc.) in BA_Eenterotypes, 2 specific genera (*Epulopiscium* and *Colinsella*) in BL_E enterotypes and 24 specific genera (*Succinivibrio*, Sporobacter, *Dietzia* and etc.) in S_E group, respectively (Fig. 5B-C). Moreover, *Pseudomonas*, *Fusobacterium*, and *Peptostreptococcus*, both in the three enterotypes and all samples (Fig. 5B and C), Among these, *Peptostreptococcus* was identified as a biomarker for CRC patients in the three enterotypes and all samples.

### Differential bacterial biomarkers in each enterotype can be used to distinguish three human cohorts based random forest classification

**Fig. 6:**
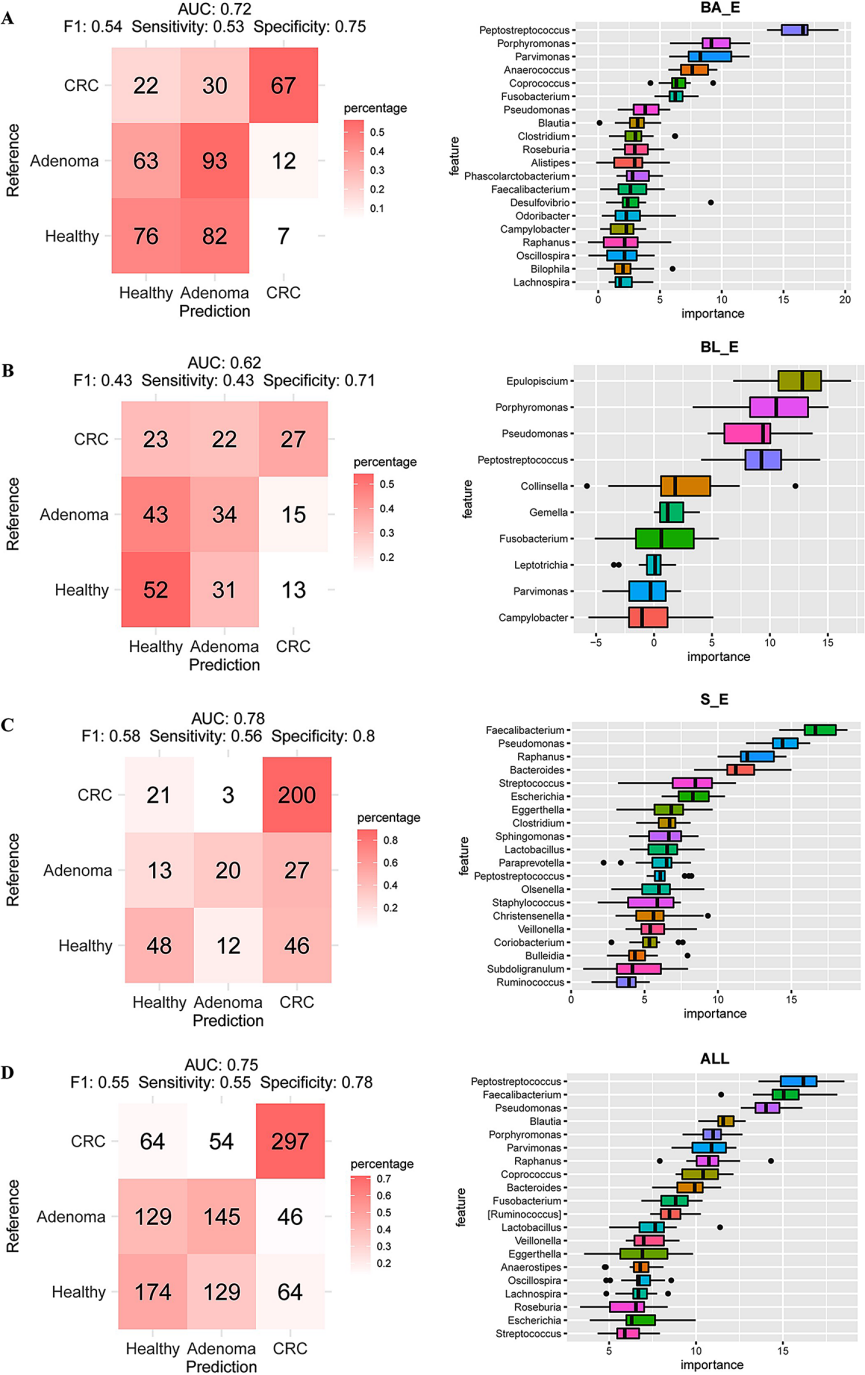
Construction of a classification model to distinguish among healthy, adenoma, and CRC based on enterotypes and all samples. **A**: Random forest classifier prediction of the top 20 characteristic bacteria in the BA_E enterotype of the three human cohorts. **B**: Random forest classifier prediction of top 20 characteristic bacteria in the BL_E enterotype of the three human cohorts. **C**: Random forest classifier prediction of the top 20 characteristic bacteria in the S_E enterotype of the three human cohorts. **D**: Random forest classifier prediction of the top 20 characteristic bacteria in the three human cohorts based on all samples

**Fig. 7:**
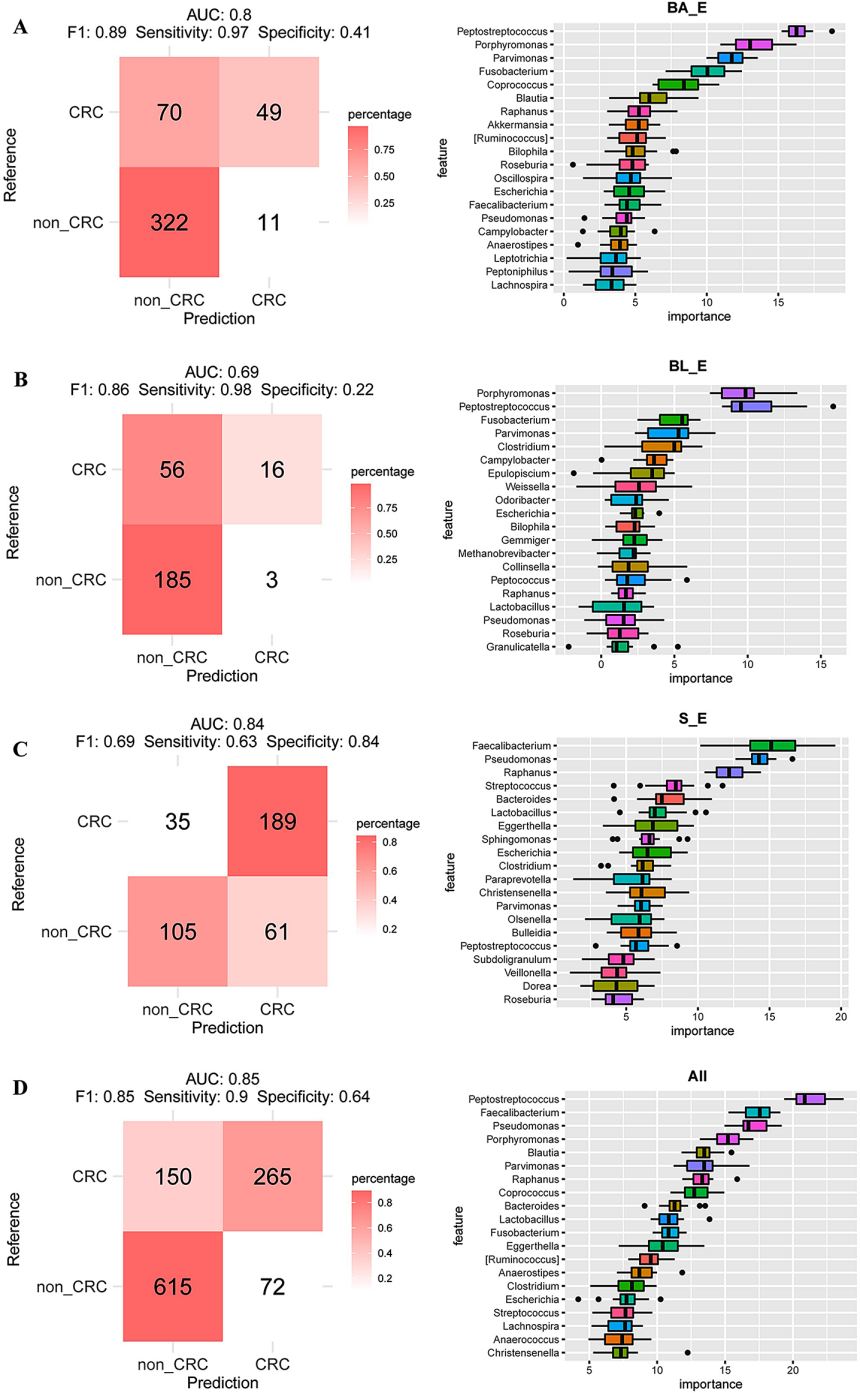
Construction of a classification model to distinguish between non-colorectal cancer and colorectal cancer samples based on enterotype and all samples. **A**: Predictive ability of the BA_E enterotype in distinguishing between non-CRC and CRC samples. **B**: Predictive ability of the BL_E enterotype in distinguishing between non-CRC and CRC samples. **C**: Predictive ability of the S_E enterotype in distinguishing between non-CRC and CRC. **D**: Predictive ability of all samples in distinguishing between non-CRC and CRC samples

Given our findings of different compositions of the three enterotypes in subject populations, we proceeded to establish whether these enterotypes have potential utility in differentiating among healthy, adenoma, and CRC subjects. Initially, we assessed the predictive ability of three-class classification in identifying healthy, adenoma, and CRC subjects. With respect to the BA_E group, the AUC of classification was 0.75 (F1 score = 0.54), with a sensitivity and specificity of 0.53 and 0.75, respectively (Fig. 6A). Furthermore, using this model, the characteristics of *Peptostreptococcus*, *Porphyromonas*, *Parvimonas*, *Anaerococcus*, and *Coprococcus* were found to have high importance scores. For the BL_E group, the AUC value was 0.62 (F1 score = 0.43), with a sensitivity and specificity of 0.43 and 0.71, respectively (Fig. 6B), and the top genera ranked in terms of importance were *Epulopiscium*, *Porphyromonas*, *Pseudomonas*, *Peptostreptococcus*, and *Collinsella*. For the S_E group, we obtained AUC, sensitivity, and specificity values of 0.78 (F1 score = 0.58), 0.56, and 0.8, respectively (Fig. 6C), and the genera *Faecalibacterium*, *Pseudomonas*, *Raphanus*, *Bacteroides*, and *Streptococcus* were assigned high importance scores. On the basis of these findings, we established that the predictive performance of S_E was superior to that of BA_E and BL_E. In addition, we also determined the predictive performance of differential bacteria without initial enterotype clustering (Fig. 6D). Using this model, we obtained an AUC value of 0.75 (F1 score = 0.55) for the classification of healthy, adenoma, and CRC subjects, with corresponding sensitivity and specificity values of 0.55 and 0.78, respectively, with *Peptostreptococcus*, *Faecalibacterium*, *Pseudomonas*, *Blautia*, and *Porphyromonas* being identified as the top ranked characteristic bacterial genera (See Fig. 7).

We also used a two-class classification model to distinguish CRC from non-CRC samples. Using this model, we obtained AUC values 0.69, 0.68, 0.79, and 0.78 for BA_E, BL_E, S_E, and all samples, respectively. Among the bacterial genera, we obtained high importance scores for *Peptostreptococcus*, *Porphyromonas*, *Parvimonas*, *Fus*obacterium, and *Coprococcus* in the BA_E model; *Porphyromonas*, *Peptostreptococcus*, *Fusobacterium*, *Parvimonas*, and *Clostridium* in the BL_E model; and *Faecalibacterium*, *Pseudomonas*, *Raphanus*, *Streptococcus*, and *Bacteroides* in the S_E model. Considering all models combined, *Peptostreptococcus*, *Faecalibacterium*, *Pseudomonas*, *Porphyromonas*, and *Blautia* were identified as the top five most important bacterial genera. Consistent with the findings obtained based on three-class classification analysis, we found that among three enterotypes, S_E showed the highest predictive performance. However, compared with our analysis based on all samples, we identified no significant advantages regarding the disease-predictive power of enterotypes. These findings were confirmed using the validation sets (Figure S5).

## Discussion

Enterotype profiling, which entails the stratification of human gut microbiota, is considered a reliable method for gaining insights into the gut microbial community, independent of age, sex, and ethnicity. However, relatively few studies have sought to examine changes in the microbial composition of enterotypes from the perspective of CRC development. In this study, we classified gut microbiota of the enrolled subjects into three enterotypes (BA_E, BL_E, and S_E) based on similarities in bacterial composition. The dominant bacteria of BA_E, BL_E, and S_E were *Bacteroide*, *Blautia*, and *Streptococcus*. We also observed that the S_E type contained a higher number of CRC samples, and that this enterotype had highest precision in distinguishing among samples obtained from healthy, adenoma, and CRC subjects, as well as between CRC and non-CRC samples.

The BA_E enterotype, characterized by the predominance of *Bacteroides*, has been confirmed to be closely associated long-term diet, particularly the composition of animal proteins, multiple amino acids, and saturated fats [13]. For this enterotype, we found that bacteria in the genera *Akkermansia* and *Gemmiger* were higher in samples obtained from CRC subjects than in those of healthy and adenoma subjects. *Akkermansia muciniphila*, a Gram-negative anaerobic bacterium from the genus *Akkermansia*, has been found to trigger host metabolic and immune responses in the intestinal mucosa and is considered an indicator of host metabolic status [29, 30]. Moreover, *A. muciniphila* has been reported to promote CRC development in mice, possibly by inducing early inflammation and promoting the proliferation of epithelial cells [31]. In addition, Osman et al. detected an enrichment of *A. muciniphila* in cancerous tissues and accordingly identified this species as a potential bacterial biomarker of CRC [32]. *Gemmiger* (a genus of budding anaerobic bacteria) was found to be enriched in early-stage hepatocellular carcinoma (HCC) compared with liver cirrhosis, and changes in the abundance of these species may contribute to HCC development [33]. However, to date there been no reports regarding its association with the development of CRC.

*Faecalibacterium*, *Roseburia*, and *Bacteroides* showed a significant enrichment in the BL_E. Among these, species of *Faecalibacterium* are butyrate-producing bacteria, the abundance of which was found to lower in patients with CRC, which is consistent with the findings of a previous study [34]. *Faecalibacterium prausnitzii* (from *Faecalibacterium*) has been reported to maintain intestinal healthy by producing energy and anti-inflammatory metabolites and is significantly associated with fatty acid synthesis pathways [35]. Liang et al. have suggested that *F. prausnitzii* is associated with mutation of the *APC* gene and may have potential utility in predicting the progression of intestinal adenomatous polyps to CRC [36]. *Roseburia* spp. is also involved in the synthesis of butyric acid and consists of obligate Gram-positive anaerobic bacteria, which may affect anti-inflammatory properties, immune maintenance, and colonic motility [37]. Previous studies showed that *Roseburia* displayed reduced abundance in the CRC patients [34, 38]. Contrastingly, in the present study, we established that the abundance of *Roseburia* was higher in CRC patients than in healthy and adenoma subjects. This may be due to the different sources of the samples. For the purposes of this study, we enrolled subjects from two datasets comprising Chinese individuals, especially the proportion of Chinese CRC patients was more than 50%. This speculation was confirmed by Geng et al. [39]. Similarly, they found that *Roseburia* was overexpressed in CRC tissues from Chinese patients. Notably, the above previous studies were conducted in subjects from Finland and African American subjects, and dietary and genetic factors may have comparatively little influence on *Roseburia* differentiation among Chinese CRC patents. However, further studies will be necessary to identify the specific causal factors. As a Gram-negative bacterium, we observed that the relative abundance of *Bacteroides* showed a gradual decreasing trend in healthy-adenoma-CRC samples. Similarly, Liang et al. [40] found that *Bacteroides* was significantly reduced in CRC patients, and it helped to improve the specificity of disease diagnosis. In this context, “driver-passenger model” can be used to reveal the role of gut microbiota in the pathogenesis of CRC, that is, CRC is initiated by “driver” bacteria that cause changes in the tumor microenvironment, allowing “passenger” bacterial colonization that can promote CRC progression [41]. A latest research found that *Bacteroides fragilis* (a member of *Bacteroides*) served a potential driving role and it was also associated with early-stage adenoma [42].

*Streptococcus*, characteristic bacteria of the S_E enterotype, were identified a biomarker of CRC based on LDA analysis. *Streptococcus bovis* (a member of *Streptococcus*) is a Gram-positive bacterium and its abundance is higher in CRC patients than in healthy controls, thereby indicating that this bacterium may play a carcinogenic role in CRC [43]. In addition, *Bacteroides* was established to be a predominant genus in this enterotype, whereas *Escherichia* was mainly enriched in adenoma samples. Both *Bacteroides fragilis* (a member of Bacteroides) and *Escherichia coli* (a member of Escherichia) can cause direct DNA damage and trigger genomic instability in cells [43, 44]. Among them, *Escherichia coli* was confirmed to be directly implicated in the development of adenomas and subsequent progression to CRC [11]. For each of the identified enterotypes, multiple microbial interactions were also observed in all three of the subject populations, thereby indicating that the combined activities of gut bacteria may contribute to altering disease states. However, the underlying mechanisms await further elucidation.

An important aspect of the present study is that we performed a stratified analysis using different enterotypes and evaluated their disease-related predictive powers. Results showed that compared with the other two enterotypes, S_E had higher predictive power in the healthy/adenoma/CRC as well as non-CRC/CRC, which was validated using metagenomic data. In the S_E group, there was an unequal distribution of samples from subjects with different disease status, which we suspect may have contributed to enhancing its predictive power. Moreover, the three identified enterotypes differed with respect to the bacteria making the high contribution, with *Peptostreptococcus*, *Epulopiscium*, and *Faecalibacterium* being assigned the highest importance scores in the BA_E, BL_E, and S_E enterotypes, respectively. *Peptostreptococcus* has previously been found to be enriched in the feces or tumor tissue obtained from patients with adenoma or CRC and was involved in CRC carcinogenesis [45]. Lin et al. [46] constructed predictive model based on 10 key species, including those in the genus *Peptostreptococcus*, which displayed the best performance in distinguishing between adenoma and CRC patients. In addition, precancerous lesions of colon cancer have been shown to be associated with changes in the abundance of *Faecalibacterium* [47]. The findings of these studies may partially explain the utility of enterotypes in distinguishing adenomas from colorectal cancer. However, whether *Epulopiscium* is involved in the transition from adenoma to cancer has yet to be ascertained. Although compared with no enterotype classification, the prediction accuracy of the model was better in the identification of healthy, adenoma, and CRC subjects based on enterotype. However, admittedly, enterotype prediction did not show a clear advantage in distinguishing CRC from non-CRC. This may have something to do with the uneven distribution of samples. In addition, there are some similarities in community structure and diversity between gut microbes in CRC patients and those in adenoma patients. This also increases the difficulty of prediction based on enterotype to some extent. Therefore, further studies are needed to further screen specific enterotype related gut microbes and verify the potential of gut type-based prediction in identifying disease states.

Our evidence provides to indicate that important changes may occur in the gut microbiota during the adenoma (early) stage of CRC and are associated with CRC progression. Moreover, we established that each of the three identified enterotypes has a unique microbial composition and to the best of our knowledge, this is the first study to identify the diagnostic potential of enterotypes in distinguishing among healthy, adenoma, and CRC subjects. Despite our important findings, however, the study does have certain limitations. Firstly, we did not assess correlations among the microbiome, clinical parameters, and enterotypes, and thus based on the data obtained, we are currently unable establish precise causal relationships between microbiota composition and disease. Secondly, the microbiota data used in this study were analyzed at the genus level, and consequently, we were unable to identify specific changes in the gut microbiota. Thirdly, there was an unequal distribution of sample sizes among the three populations with respect to the different enterotypes, which may have unintentionally biased disease prediction. Accordingly, the findings of this study should be interpreted conservatively.

## Conclusion

In summary, enterotype stratification of 1102 subjects was performed based on 16 S rRNA sequencing data. We identified three enterotypes driven by *Bacteroide*, *Blautia*, and *Streptococcus*, respectively, which were characterized by clear differences in microbial composition and the relative abundances of certain bacteria in samples obtained from healthy, adenoma, and CRC subjects. Moreover, using these enterotypes, we constructed predictive models for distinguishing among healthy, adenoma, and CRC subjects, thereby contributing to our understanding of the interaction among enterotypes and their influence on CRC development.

### Electronic supplementary material

Below is the link to the electronic supplementary material.

Supplementary Material 1

## Data Availability

The datasets generated for this study can be accessed from the NCBI Sequence Read Archive (SRA) database under the accession number PRJNA913403 (http://www.ncbi.nlm.nih.gov/bioproject/913403), PRJNA913257 (http://www.ncbi.nlm.nih.gov/bioproject/913257) and PRJNA913399 (http://www.ncbi.nlm.nih.gov/bioproject/913399 ).
