# Supplementary material for: Identification of enterotype and its predictive value for patients with colorectal cancer
Source: Gut Pathog. 2024 Feb 27;16:12. doi: 10.1186/s13099-024-00606-y (PMC10897996; doi:10.1186/s13099-024-00606-y)

**Figure S1.** **The top eight bacterial genera in the three enterotypes based on healthy, adenoma, and CRC samples.**


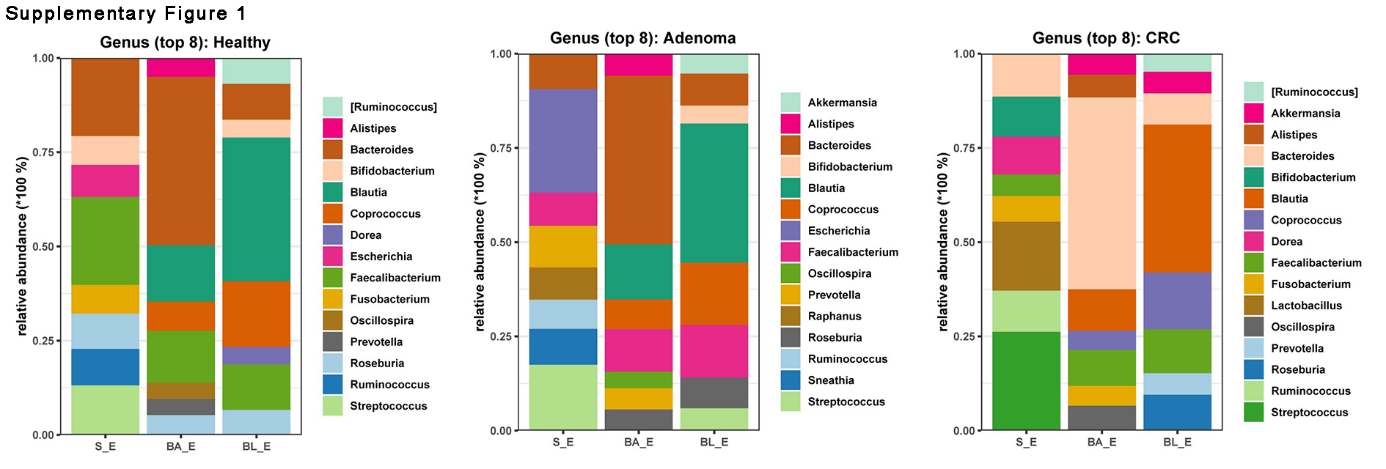


**Figure S2.** **Correlations between differential genera** **within the BA_E enterotype in the healthy,** **adenoma, and CRC samples.**

A: Correlation plot of gut microbiota in healthy individuals. B: Correlation plot of gut microbiota in patients with adenoma. C: Correlation plot of gut microbiota in patients with CRC.


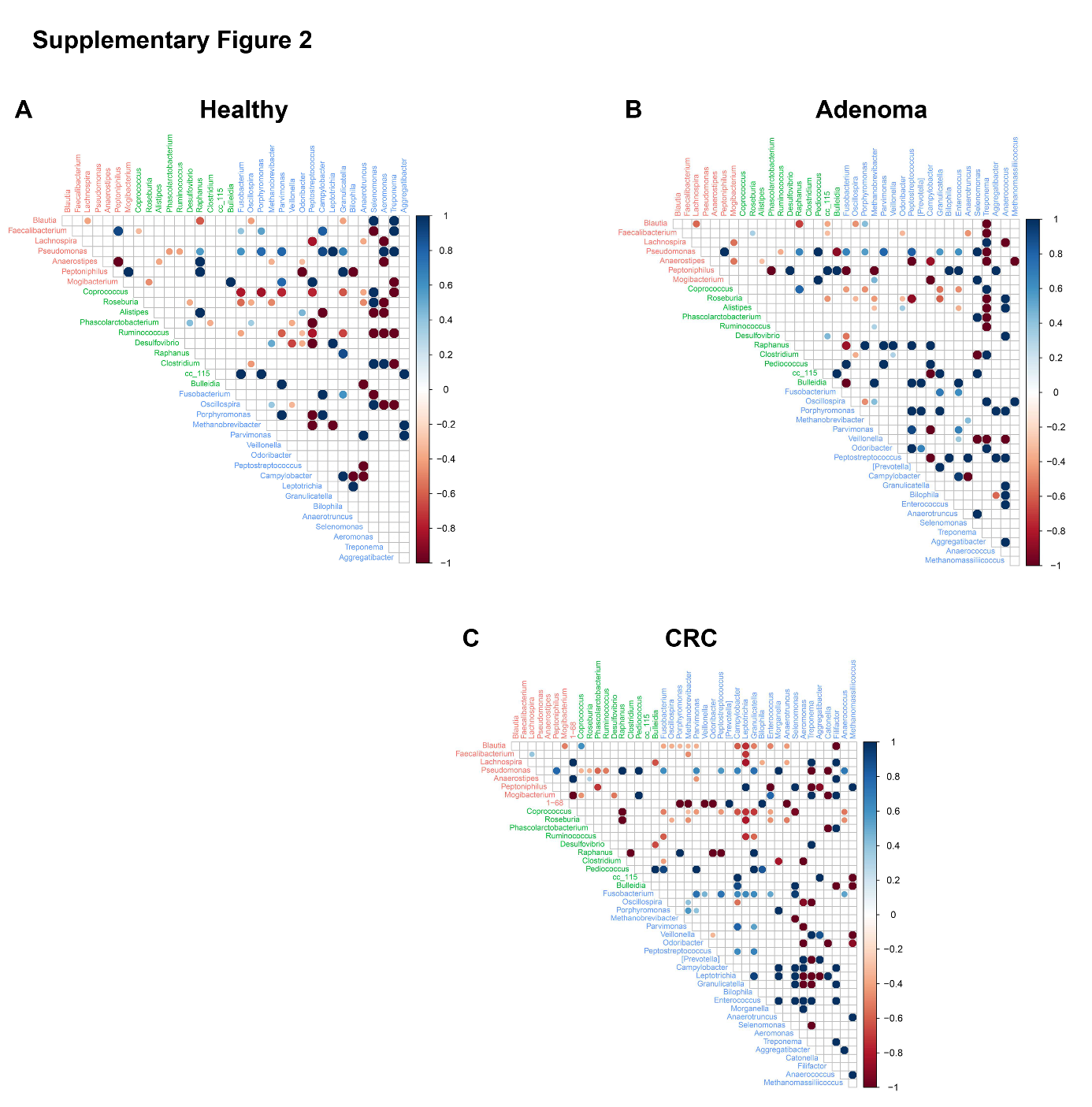


**Figure S3.** **Correlations between differential genera within** **the BL_E enterotype in the healthy, adenoma, and CRC samples.**

A: Correlation plot of gut microbiota in healthy individuals. B: Correlation plot of gut microbiota in patients with adenoma. C: Correlation plot of gut microbiota in patients with CRC.


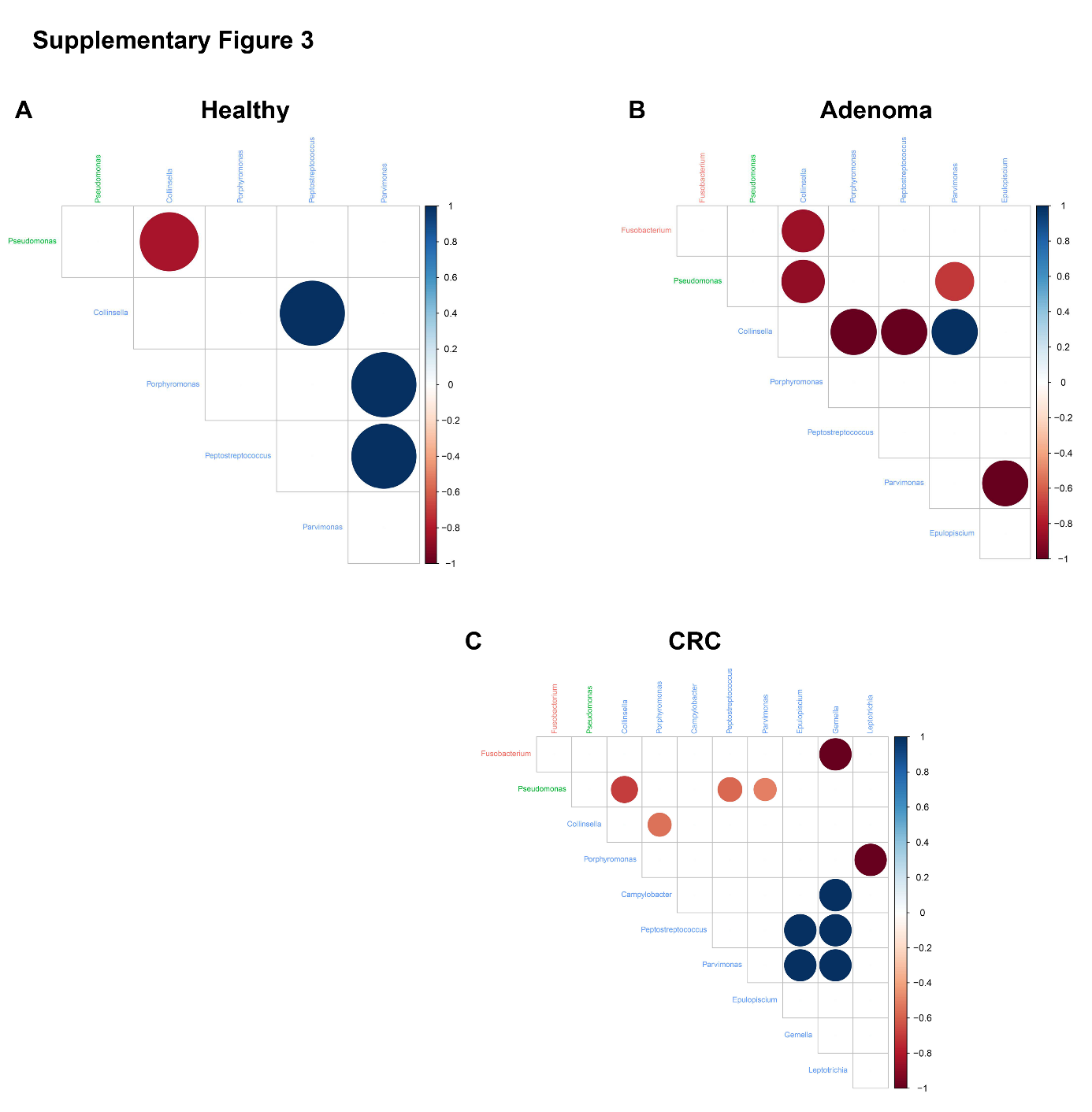


**Figure S4. The correlation analysis between differential genera in the healthy, adenoma, and CRC within S_E type.**

A: Correlation plot of gut microbiota in healthy individuals. B: Correlation plot of gut microbiota in patients with adenoma. C: Correlation plot of gut microbiota in patients with CRC.


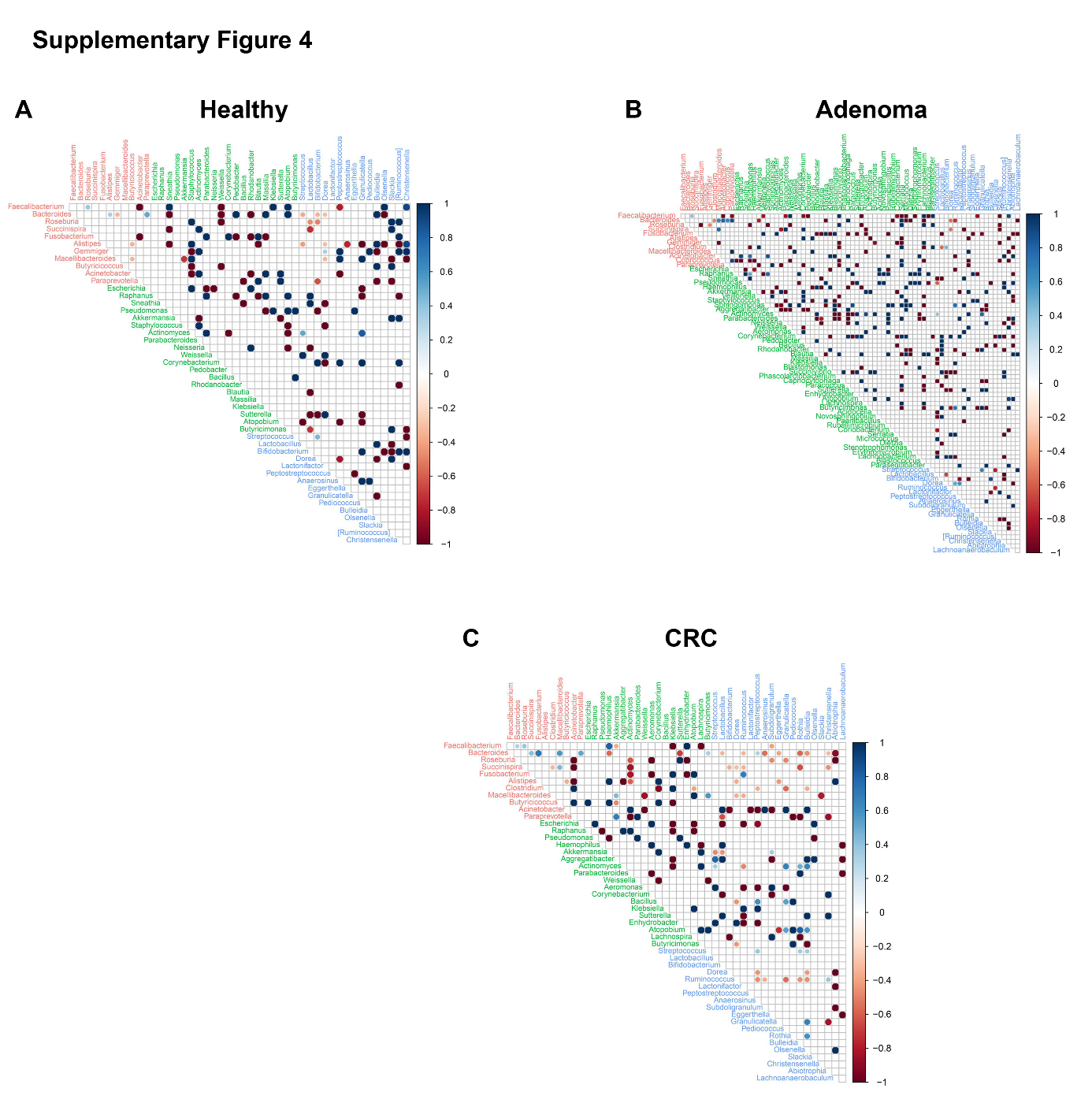


**Figure S5. Predictive performance of the model was validated using metagenomic data.**


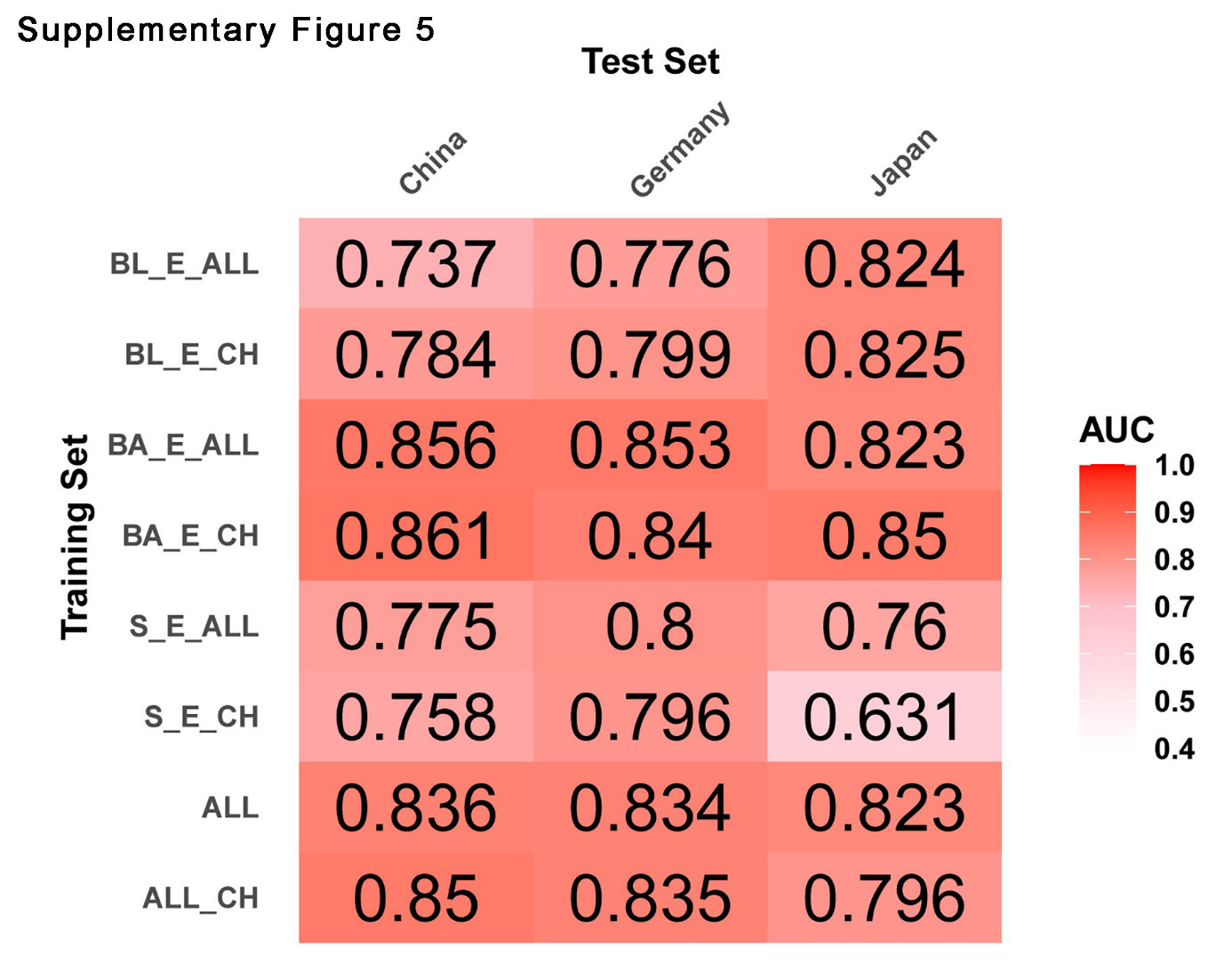

Supplement: Supplementary file 1 — Supplementary Material 1 [file 13099_2024_606_MOESM1_ESM.docx]
